# MKK3 as oncotarget

**DOI:** 10.18632/aging.100878

**Published:** 2016-01-21

**Authors:** Gianluca Bossi

**Affiliations:** Experimental Oncology Laboratories, and Laboratory of Medical Physics and Expert Systems, Regina Elena National Cancer Institute, Rome, Italy

**Keywords:** mitogen-activated protein kinase, RNA interference, gene targeting, tumour therapy, drugs dose reduction

The mitogen-activated protein kinase kinase 3 (MAP2K3, MKK3) is a member of the dual-specificity protein kinase group (MKK) that belongs to the mitogen-activated protein kinase (MAPK) signaling pathway. Following different forms of stressful stimuli and inflammatory cytokines, MKK3 is activated through phosphorylation on serine and threonine residues at sites Ser189 and Thr193 by MKKK proteins (MEKK 1-4) [1]. Activated MKK3 phosphorylates specifically p38MAPK, an important kinase involved in a plethora of cellular programs, including cell differentiation, motility, division, and death. Consistent literature identified MKK3 as relevant player involved in tumor invasion and progression in gliomas and breast tumors.

We previously identified MKK3 in a gene expression profile array performed with p53-null H1299 cells ectopically expressing mutant p53-R175H gain-of-function (GOF), one of the most frequent alteration revealed in human cancer [2]. Studies of validation performed with a panel of cancer cell lines harbouring different p53 mutants (R273H, R280K) showed the generalized effect since other p53 mutants were able to upregulated MKK3 gene expression. By contrast, normal wild type (wt) p53 protein does not affect the MKK3 expression [3]. Mechanistically, we demonstrate that mutp53 is physically recruited on MKK3 promoter and that through the involvement of specific transcriptional co-factors (NF-κB and NF-Y) sustains MKK3 expression [3]. To explore whether MKK3 might contribute in mutp53 GOF activities, functional studies were performed by silencing the endogenous MKK3 protein by using inducible RNA interference (RNAi) system. We demonstrated the MKK3 depletion impairs at different extent the cell proliferation and cell survival of tested mutp53 cancer cells (HT29, MDA-MB468, MDA-MB231, SKBR3). The effects were not mutp53 cell-context dependent since observed also p53-null (H1299) human cancer cells predicting that MKK3 might represent a generally required factor [3]. We further extended our studies also in other cell lines in wtp53 cell-context with transformed and not-transformed phenotype. Interestingly, results beside confirming MKK3 as generally required factor further suggested MKK3 as tumor-specific required factor. Indeed, similarly to mutp53 cancer cells, MKK3 depletion affects cell proliferation and cell survival also in wtp53 carrying cancer cells (MCF7, HCT116) but not in primary no-transformed cells (FB1329, MCF10A) [4]. Under microscope examination MKK3 depletion induces large cytoplasmic vacuoles in cancer but not normal cells. Accordingly, we investigated whether MKK3 silencing might induce autophagic degeneration. Through biochemical analyses we found that MKK3 depletion induces accumulation of LC3-II levels and SQSTM1/p62 degradation in both wt and mutp53 carrying cancer cells. Moreover, MKK3 depletion raises wtp53 protein level correlated to endoplasmic reticulum (ER) stress in MCF7 and HCT116 cancer cells, and mutp53 degradation in HT29 and MDA-MB468 cancer cells. Chloroquine (CQ) treatment, an inhibitor of final stage of autophagy, rescues mutp53 proteins levels strongly suggesting that autophagy may have major roles in mutp53 protein reduction. Furthermore, MKK3 depletion induces autophagic cell death in studied cancer cells since either CQ treatment or ATG5 (autophagy protein 5) depletion rescued significantly the death fraction [4]. The successful cancer eradication often needs the combination of different anticancer strategies to overcome chemoresistance and/or improve chemosensitivity. Therefore, we explored whether the MKK3 targeting might constitute a novel strategy to boost tumor cell response to anticancer drug. We demonstrated that MKK3 depletion improves response to therapy in both wt and mutp53 cancer cells allowing chemotherapeutic-dose-reduction. Indeed, we demonstrate that MKK3 depletion in co-treatment with lower ADR dose (1,0 μM) induce significantly higher PARP cleavage with respect to control cells challenged with higher ADR dose (2,0 μM) in both wt and mutp53 cancer cells (HCT116 and HT29) [4]. Similar results were achieved with 5-fluorouracil (5-FU). Indeed control HT29 cells challenged with higher dose of 5-FU (10 μM) revealed a colony forming ability similar to that of MKK3 depleted cells co-treated with 10 times lower 5-FU dose (1,0 μM) [4]. Additionally, MKK3 targeting combined to 5-FU scheduled treatments (50mg/kg, intraperitoneal) inhibit significantly xenograft tumor growth when compared to controls tumor bearing mice [4].

Our results are in agreement with other studies demonstrating that MAPK14/p38 MAPK is required for cell proliferation and survival and its inhibition leads to cell cycle arrest and autophagy-mediated cell death in colorectal tumors [5]. Other work demonstrated that p38 MAPK inhibition cooperates with cisplatin to kill breast and colon cancer cells *in vitro* and *in vivo*, and with doxorubicin and sorafenib in lung and liver cancer respectively [6] However, given the plethora of functions that the p38 MAPK pathway can perform, especially in normal cells, its inhibition need to be carefully evaluated. Indeed, of interest, a study performed in non-small cell lung cancer (NSCLC) and head and neck squamous cell carcinoma cell lines revealed the altered balance between the MKK3/MKK6 (another p38 MAPK specific activator) as novel mechanism of resistance to cisplatin (cDDP). In the suggested scenario high MKK3 levels mediate constitutive hyper-activation of the p38 MAPK pathway which is linked to cDDP resistance [7].

In summary the results obtained by our group, according to other studies, are suggesting MKK3 as oncotarget. Indeed, its inhibition, suggestively deleterious for cancer but not normal primary cells, might constitute a novel potential and promising therapeutic strategy, that in alternative to the p38 MAPK inhibition, could potentiate the efficiency of chemotherapies in non-responder patients.

## References

[1] Dérijard B (1995). Science.

[2] Bossi G (2008). Cell Cycle.

[3] Gurtner A (2010). J Biol Chem.

[4] Baldari S (2015). Cell Death Dis.

[5] Chiacchiera F (2008). Cancer Lett.

[6] Pereira L (2013). Embo Mol Med.

[7] Galan-Moya EM (2011). PLoS One.

